# 2-(4-Fluoro­phen­yl)-*N*-{4-[6-(4-fluoro­phen­yl)-2,3-dihydro­imidazo[2,1-*b*][1,3]thia­zol-5-yl]pyridin-2-yl}acetamide

**DOI:** 10.1107/S1600536810012766

**Published:** 2010-04-21

**Authors:** Roland Selig, Dieter Schollmeyer, Wolfgang Albrecht, Stefan Laufer

**Affiliations:** a Eberhard-Karls-University Tübingen, Auf der Morgenstelle 8, 72076 Tübingen, Germany; b University Mainz, Duesbergweg 10-14, 55099 Mainz, Germany; c c-a-i-r biosciences GmbH, Paul-Ehrlich-Strasse 15, 72076 Tübingen, Germany

## Abstract

In the crystal structure of the title compound, C_24_H_18_F_2_N_4_OS, the imidazole system makes dihedral angles of 34.3 (1) and 43.9 (1)°, respectively, with the directly attached 4-fluoro­phenyl and pyridine rings. The crystal structure is stabilized by inter­molecular N—H⋯N hydrogen bonding and by an intra­molecular C—H⋯O hydrogen inter­action. The F atom of the 2-(4-fluoro­phen­yl) group is disordered over two positions with site-occupancy factors of 0.75 and 0.25.

## Related literature

For related compounds and their biological relevance, see: Ziegler *et al.* (2009 ▶).
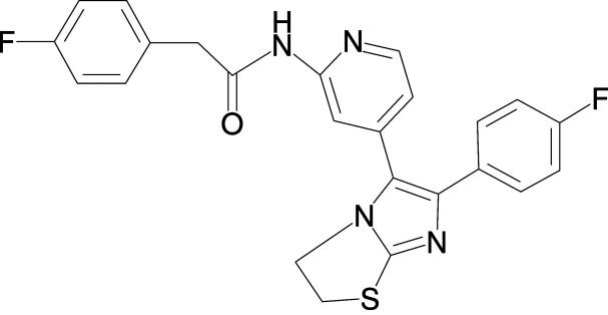


## Experimental

### 

#### Crystal data

C_24_H_18_F_2_N_4_OS
*M* *_r_* = 448.48
Monoclinic, 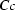

*a* = 4.9179 (3) Å
*b* = 23.592 (1) Å
*c* = 18.4834 (9) Å
β = 91.523 (2)°
*V* = 2143.8 (2) Å^3^
*Z* = 4
Mo *K*α radiation
μ = 0.19 mm^−1^
*T* = 173 K
0.35 × 0.16 × 0.08 mm

#### Data collection

Bruker SMART APEXII diffractometer
10277 measured reflections
4846 independent reflections
4129 reflections with *I* > 2σ(*I*)
*R*_int_ = 0.028

#### Refinement

*R*[*F* ^2^ > 2σ(*F* ^2^)] = 0.038
*wR*(*F* ^2^) = 0.090
*S* = 1.03
4846 reflections
298 parameters
2 restraints
H-atom parameters constrained
Δρ_max_ = 0.23 e Å^−3^
Δρ_min_ = −0.20 e Å^−3^
Absolute structure: Flack (1983 ▶), 2197 Friedel pairs
Flack parameter: 0.07 (6)

### 

Data collection: *APEX2* (Bruker, 2006 ▶); cell refinement: *SAINT* (Bruker, 2006 ▶); data reduction: *SAINT*; program(s) used to solve structure: *SIR97* (Altomare *et al.*, 1999 ▶); program(s) used to refine structure: *SHELXL97* (Sheldrick, 2008 ▶); molecular graphics: *PLATON* (Spek, 2009 ▶); software used to prepare material for publication: *PLATON*.

## Supplementary Material

Crystal structure: contains datablocks I, global. DOI: 10.1107/S1600536810012766/im2189sup1.cif

Structure factors: contains datablocks I. DOI: 10.1107/S1600536810012766/im2189Isup2.hkl

Additional supplementary materials:  crystallographic information; 3D view; checkCIF report

## Figures and Tables

**Table 1:** Hydrogen-bond geometry (Å, °)

*D*—H⋯*A*	*D*—H	H⋯*A*	*D*⋯*A*	*D*—H⋯*A*
N13—H13⋯N6^i^	0.97	2.02	2.980 (2)	171
C8—H8⋯O15	0.95	2.24	2.845 (3)	120

Symmetry code: (i) 
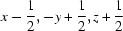
.

**Fig. 1.**
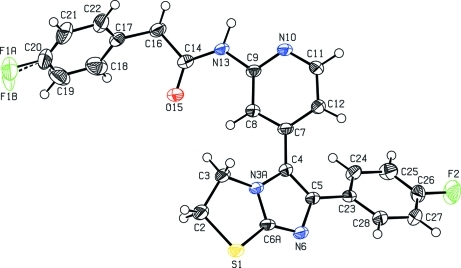
View of compound I. Displacement ellipsoids are drawn at the 50% probability level.

## supplementary crystallographic information

### Comment

SKF86002 was an early lead compound of many imidazole based p38 MAP kinase inhibitors. A further improvement of these inhibitors was the modification of the pyridyl moiety by substitution with amines in C-2 position of the pyridine. This donor acceptor system can interact with hinge region/MET109 by bidentate hydrogen bonding. Additional interactions of the attached residues with the hydrophobic region II strongly increases potency of these compounds (Ziegler *et al.* 2009).

The imidazole system of the title compound 2-(4-fluorophenyl)-*N*-{4-[6-(4-fluorophenyl)-2,3-dihydroimidazo[2,1-b][1,3] thiazol-5-yl]-pyridin-2-yl}acetamide, C_24_H_18_F_2_N_4_OS, makes dihedral angles of 34.3 (1)° and 43.9 (1)° with the directly attached 4-fluorophenyl and the pyridine rings, respectively. The crystal structure is characterized by an intermolecular hydrogen bond N13—H13···N6 (2.02 Å). The molecular conformation is stabilized by an intramolecular C8—H8···O15 (2.24 Å) interaction. The flourine atom F1 is disordered over two positions with s.o.f 0.75:0.25.

### Experimental

4-fluorphenylacetic acid (296 mg) was dissolved in 3 ml *N*-methylpyrrolidinone. After addition of 332 mg of carbonyldiimidazole the mixture was stirred for 1 h at room temperature. 200 mg 4-[6-(4-fluorophenyl)-2,3-dihydroimidazo[2,1-b][1,3]thiazol-5-yl]pyridin-2-amine was added and the reaction mixture was heated to 383 K for 19 h. The reaction mixture was quenched with a solution of concentrated sodium hydrogen carbonate and extracted with ethylacetate. The crude product was purified by flash chromatography (eluent: ethylacetate/hexane 2/1) to yield 155 mg (54%) of the title compound. Crystals suitable for X-ray analysis were obtained by slow crystallization from methanol at room temperature.

### Refinement

Hydrogen atoms attached to carbons were placed at calculated positions with C—H = 0.95 Å (aromatic) or 0.98–0.99 Å (*sp*^3^ C-atom). All H atoms were refined in the riding-model approximation with isotropic displacement parameters (set at 1.2–1.5 times of the *U*_eq_ of the parent atom). The flourine F1 is disordered over two positions with s.o.f 0.75:0.25.

### Crystal data

**Table:**

C_24_H_18_F_2_N_4_OS	*F*(000) = 928
*M**_r_* = 448.48	*D*_x_ = 1.390 Mg m^−^^3^
Monoclinic, *C**c*	Mo *K*α radiation, λ = 0.71073 Å
Hall symbol: C -2yc	Cell parameters from 3757 reflections
*a* = 4.9179 (3) Å	θ = 2.2–26.4°
*b* = 23.592 (1) Å	µ = 0.19 mm^−^^1^
*c* = 18.4834 (9) Å	*T* = 173 K
β = 91.523 (2)°	Plate, yellow
*V* = 2143.8 (2) Å^3^	0.35 × 0.16 × 0.08 mm
*Z* = 4	

### Data collection

**Table:**

Bruker SMART APEXII diffractometer	4129 reflections with *I* > 2σ(*I*)
Radiation source: sealed tube	*R*_int_ = 0.028
graphite	θ_max_ = 28.2°, θ_min_ = 1.7°
CCD scan	*h* = −6→6
10277 measured reflections	*k* = −30→28
4846 independent reflections	*l* = −23→24

### Refinement

**Table:**

Refinement on *F*^2^	Secondary atom site location: difference Fourier map
Least-squares matrix: full	Hydrogen site location: inferred from neighbouring sites
*R*[*F*^2^ > 2σ(*F*^2^)] = 0.038	H-atom parameters constrained
*wR*(*F*^2^) = 0.090	*w* = 1/[σ^2^(*F*_o_^2^) + (0.0433*P*)^2^ + 0.4891*P*] where *P* = (*F*_o_^2^ + 2*F*_c_^2^)/3
*S* = 1.02	(Δ/σ)_max_ = 0.001
4846 reflections	Δρ_max_ = 0.23 e Å^−^^3^
298 parameters	Δρ_min_ = −0.20 e Å^−^^3^
2 restraints	Absolute structure: Flack (1983), 2197 Friedel pairs
Primary atom site location: structure-invariant direct methods	Flack parameter: 0.07 (6)

### Special details

**Table:**

Geometry. All esds (except the esd in the dihedral angle between two l.s. planes) are estimated using the full covariance matrix. The cell esds are taken into account individually in the estimation of esds in distances, angles and torsion angles; correlations between esds in cell parameters are only used when they are defined by crystal symmetry. An approximate (isotropic) treatment of cell esds is used for estimating esds involving l.s. planes.
Refinement. Refinement of *F*^2^ against ALL reflections. The weighted *R*-factor wR and goodness of fit *S* are based on *F*^2^, conventional *R*-factors *R* are based on *F*, with *F* set to zero for negative *F*^2^. The threshold expression of *F*^2^ > σ(*F*^2^) is used only for calculating *R*-factors(gt) etc. and is not relevant to the choice of reflections for refinement. *R*-factors based on *F*^2^ are statistically about twice as large as those based on *F*, and *R*- factors based on ALL data will be even larger.

### Fractional atomic coordinates and isotropic or equivalent isotropic displacement parameters (Å^2^)

**Table:**

	*x*	*y*	*z*	*U*_iso_*/*U*_eq_	Occ. (<1)
S1	0.82180 (11)	0.16231 (2)	−0.12400 (3)	0.03583 (14)	
F1A	−0.3136 (9)	−0.09848 (19)	0.2022 (2)	0.0837 (13)	0.75
F1B	−0.454 (3)	−0.0837 (5)	0.1902 (9)	0.086 (4)	0.25
F2	0.7391 (4)	0.53748 (6)	0.01213 (10)	0.0609 (4)	
C2	0.6645 (6)	0.11864 (10)	−0.05475 (12)	0.0420 (6)	
H2A	0.4683	0.1135	−0.0662	0.050*	
H2B	0.7517	0.0808	−0.0525	0.050*	
C3	0.7039 (6)	0.14935 (9)	0.01721 (11)	0.0386 (6)	
H3A	0.5578	0.1394	0.0508	0.046*	
H3B	0.8821	0.1398	0.0402	0.046*	
N3A	0.6911 (4)	0.20911 (7)	−0.00241 (9)	0.0294 (4)	
C4	0.6770 (4)	0.26014 (9)	0.03519 (10)	0.0277 (4)	
C5	0.7275 (4)	0.30126 (9)	−0.01557 (10)	0.0266 (4)	
N6	0.7779 (4)	0.27699 (7)	−0.08266 (8)	0.0302 (4)	
C6A	0.7576 (5)	0.22229 (9)	−0.07094 (10)	0.0301 (4)	
C7	0.6335 (4)	0.26246 (8)	0.11346 (10)	0.0264 (4)	
C8	0.4394 (4)	0.22883 (9)	0.14539 (10)	0.0265 (4)	
H8	0.3224	0.2053	0.1167	0.032*	
C9	0.4195 (4)	0.23019 (9)	0.22064 (10)	0.0251 (4)	
N10	0.5728 (4)	0.26393 (7)	0.26344 (9)	0.0289 (4)	
C11	0.7533 (5)	0.29740 (9)	0.23135 (11)	0.0313 (5)	
H11	0.8601	0.3222	0.2610	0.038*	
C12	0.7934 (4)	0.29797 (9)	0.15769 (10)	0.0281 (4)	
H12	0.9266	0.3220	0.1375	0.034*	
N13	0.2423 (4)	0.19595 (7)	0.25989 (8)	0.0269 (4)	
H13	0.2720	0.2071	0.3102	0.032*	
C14	0.0701 (4)	0.15550 (9)	0.23346 (11)	0.0289 (4)	
O15	0.0457 (4)	0.14302 (8)	0.16963 (8)	0.0429 (4)	
C16	−0.0944 (5)	0.12612 (10)	0.29138 (11)	0.0328 (5)	
H16A	0.0147	0.1247	0.3371	0.039*	
H16B	−0.2605	0.1485	0.3002	0.039*	
C17	−0.1743 (5)	0.06690 (10)	0.26956 (11)	0.0339 (5)	
C18	−0.3773 (6)	0.05731 (15)	0.21804 (14)	0.0533 (7)	
H18	−0.4780	0.0881	0.1979	0.064*	
C19	−0.4342 (8)	0.00127 (19)	0.19545 (17)	0.0757 (12)	
H19	−0.5744	−0.0061	0.1604	0.091*	
C20	−0.2856 (9)	−0.04181 (15)	0.22459 (18)	0.0753 (12)	
C21	−0.0855 (9)	−0.03416 (13)	0.27436 (18)	0.0693 (10)	
H21	0.0149	−0.0654	0.2934	0.083*	
C22	−0.0297 (6)	0.02066 (11)	0.29714 (14)	0.0496 (7)	
H22	0.1109	0.0268	0.3325	0.060*	
C23	0.7289 (4)	0.36344 (8)	−0.00730 (10)	0.0254 (4)	
C24	0.5520 (5)	0.39102 (9)	0.03857 (11)	0.0312 (5)	
H24	0.4270	0.3695	0.0656	0.037*	
C25	0.5560 (5)	0.44968 (10)	0.04538 (12)	0.0379 (5)	
H25	0.4361	0.4684	0.0770	0.045*	
C26	0.7373 (5)	0.47988 (9)	0.00533 (12)	0.0371 (5)	
C27	0.9136 (5)	0.45469 (10)	−0.04073 (12)	0.0383 (5)	
H27	1.0359	0.4768	−0.0680	0.046*	
C28	0.9095 (5)	0.39598 (9)	−0.04675 (11)	0.0320 (5)	
H28	1.0316	0.3777	−0.0782	0.038*	

### Atomic displacement parameters (Å^2^)

**Table:**

	*U*^11^	*U*^22^	*U*^33^	*U*^12^	*U*^13^	*U*^23^
S1	0.0618 (4)	0.0249 (2)	0.0210 (2)	−0.0009 (3)	0.0078 (2)	−0.0022 (2)
F1A	0.123 (4)	0.061 (3)	0.068 (2)	−0.052 (2)	0.019 (2)	−0.0149 (17)
F1B	0.110 (10)	0.033 (5)	0.113 (9)	−0.036 (6)	−0.005 (8)	−0.016 (5)
F2	0.0845 (12)	0.0243 (7)	0.0745 (11)	−0.0014 (8)	0.0124 (9)	−0.0004 (7)
C2	0.0714 (18)	0.0268 (11)	0.0284 (11)	−0.0066 (11)	0.0095 (11)	0.0002 (9)
C3	0.0685 (17)	0.0239 (11)	0.0234 (10)	−0.0072 (11)	0.0050 (10)	0.0033 (8)
N3A	0.0477 (11)	0.0230 (8)	0.0177 (7)	−0.0027 (8)	0.0049 (7)	0.0016 (6)
C4	0.0364 (11)	0.0260 (10)	0.0209 (9)	−0.0048 (9)	0.0041 (8)	0.0005 (7)
C5	0.0344 (11)	0.0265 (10)	0.0191 (9)	−0.0031 (9)	0.0051 (8)	−0.0002 (7)
N6	0.0458 (11)	0.0254 (9)	0.0196 (8)	−0.0018 (8)	0.0061 (7)	0.0011 (7)
C6A	0.0454 (12)	0.0294 (11)	0.0158 (8)	−0.0029 (10)	0.0046 (8)	−0.0009 (8)
C7	0.0362 (11)	0.0262 (11)	0.0170 (9)	0.0029 (9)	0.0032 (8)	0.0011 (7)
C8	0.0326 (11)	0.0267 (10)	0.0201 (9)	−0.0020 (9)	0.0025 (8)	−0.0009 (7)
C9	0.0321 (11)	0.0240 (10)	0.0194 (9)	0.0031 (8)	0.0056 (8)	0.0002 (7)
N10	0.0414 (11)	0.0277 (9)	0.0179 (7)	−0.0015 (8)	0.0048 (7)	−0.0015 (6)
C11	0.0441 (13)	0.0263 (10)	0.0235 (9)	−0.0045 (9)	0.0013 (9)	−0.0043 (8)
C12	0.0356 (11)	0.0241 (10)	0.0249 (9)	−0.0017 (9)	0.0046 (8)	0.0001 (8)
N13	0.0374 (10)	0.0265 (9)	0.0172 (7)	−0.0021 (8)	0.0071 (7)	0.0009 (6)
C14	0.0314 (11)	0.0313 (11)	0.0240 (9)	0.0022 (9)	0.0015 (8)	0.0049 (8)
O15	0.0533 (10)	0.0536 (11)	0.0219 (7)	−0.0213 (8)	0.0002 (7)	0.0032 (7)
C16	0.0371 (12)	0.0358 (12)	0.0259 (10)	−0.0019 (9)	0.0075 (8)	0.0047 (8)
C17	0.0352 (12)	0.0410 (13)	0.0260 (9)	−0.0113 (10)	0.0089 (8)	0.0041 (9)
C18	0.0392 (14)	0.080 (2)	0.0405 (14)	−0.0095 (14)	0.0002 (11)	−0.0043 (13)
C19	0.069 (2)	0.118 (3)	0.0396 (15)	−0.054 (2)	0.0048 (15)	−0.0204 (18)
C20	0.117 (3)	0.058 (2)	0.0525 (18)	−0.054 (2)	0.027 (2)	−0.0082 (15)
C21	0.112 (3)	0.0334 (15)	0.0633 (18)	−0.0212 (16)	0.0105 (19)	0.0129 (13)
C22	0.0641 (18)	0.0387 (14)	0.0457 (14)	−0.0164 (13)	−0.0073 (12)	0.0132 (11)
C23	0.0330 (10)	0.0254 (10)	0.0177 (8)	−0.0011 (9)	−0.0014 (7)	0.0018 (7)
C24	0.0357 (12)	0.0290 (11)	0.0291 (10)	−0.0002 (9)	0.0056 (9)	0.0063 (8)
C25	0.0435 (13)	0.0351 (12)	0.0352 (12)	0.0086 (10)	0.0053 (10)	0.0003 (9)
C26	0.0521 (14)	0.0205 (10)	0.0384 (12)	−0.0024 (10)	−0.0036 (10)	0.0018 (9)
C27	0.0468 (14)	0.0334 (12)	0.0348 (11)	−0.0126 (10)	0.0026 (10)	0.0074 (9)
C28	0.0381 (13)	0.0320 (12)	0.0263 (10)	−0.0046 (9)	0.0058 (9)	−0.0004 (8)

### Geometric parameters (Å, °)

**Table:**

S1—C6A	1.755 (2)	N13—C14	1.358 (3)
S1—C2	1.831 (2)	N13—H13	0.9732
F1A—C20	1.405 (5)	C14—O15	1.219 (2)
F1B—C20	1.429 (13)	C14—C16	1.526 (3)
F2—C26	1.365 (2)	C16—C17	1.503 (3)
C2—C3	1.522 (3)	C16—H16A	0.9900
C2—H2A	0.9900	C16—H16B	0.9900
C2—H2B	0.9900	C17—C18	1.380 (3)
C3—N3A	1.457 (3)	C17—C22	1.391 (4)
C3—H3A	0.9900	C18—C19	1.412 (5)
C3—H3B	0.9900	C18—H18	0.9500
N3A—C6A	1.353 (2)	C19—C20	1.355 (6)
N3A—C4	1.393 (3)	C19—H19	0.9500
C4—C5	1.377 (3)	C20—C21	1.341 (5)
C4—C7	1.469 (3)	C21—C22	1.385 (4)
C5—N6	1.394 (2)	C21—H21	0.9500
C5—C23	1.475 (3)	C22—H22	0.9500
N6—C6A	1.313 (3)	C23—C24	1.392 (3)
C7—C8	1.385 (3)	C23—C28	1.394 (3)
C7—C12	1.398 (3)	C24—C25	1.390 (3)
C8—C9	1.397 (3)	C24—H24	0.9500
C8—H8	0.9500	C25—C26	1.373 (3)
C9—N10	1.340 (3)	C25—H25	0.9500
C9—N13	1.405 (3)	C26—C27	1.367 (4)
N10—C11	1.338 (3)	C27—C28	1.390 (3)
C11—C12	1.381 (3)	C27—H27	0.9500
C11—H11	0.9500	C28—H28	0.9500
C12—H12	0.9500		
			
C6A—S1—C2	88.70 (10)	O15—C14—C16	121.9 (2)
C3—C2—S1	107.26 (15)	N13—C14—C16	113.82 (17)
C3—C2—H2A	110.3	C17—C16—C14	111.93 (17)
S1—C2—H2A	110.3	C17—C16—H16A	109.2
C3—C2—H2B	110.3	C14—C16—H16A	109.2
S1—C2—H2B	110.3	C17—C16—H16B	109.2
H2A—C2—H2B	108.5	C14—C16—H16B	109.2
N3A—C3—C2	103.86 (16)	H16A—C16—H16B	107.9
N3A—C3—H3A	111.0	C18—C17—C22	118.5 (3)
C2—C3—H3A	111.0	C18—C17—C16	121.1 (2)
N3A—C3—H3B	111.0	C22—C17—C16	120.2 (2)
C2—C3—H3B	111.0	C17—C18—C19	119.4 (3)
H3A—C3—H3B	109.0	C17—C18—H18	120.3
C6A—N3A—C4	106.57 (16)	C19—C18—H18	120.3
C6A—N3A—C3	116.46 (17)	C20—C19—C18	119.0 (3)
C4—N3A—C3	135.62 (17)	C20—C19—H19	120.5
C5—C4—N3A	104.88 (17)	C18—C19—H19	120.5
C5—C4—C7	132.72 (19)	C21—C20—C19	123.3 (3)
N3A—C4—C7	122.28 (17)	C21—C20—F1A	113.2 (4)
C4—C5—N6	110.87 (18)	C19—C20—F1A	123.3 (4)
C4—C5—C23	129.10 (17)	C21—C20—F1B	143.6 (7)
N6—C5—C23	120.02 (16)	C19—C20—F1B	92.4 (6)
C6A—N6—C5	103.95 (15)	C20—C21—C22	118.0 (3)
N6—C6A—N3A	113.68 (17)	C20—C21—H21	121.0
N6—C6A—S1	133.22 (15)	C22—C21—H21	121.0
N3A—C6A—S1	112.93 (15)	C21—C22—C17	121.7 (3)
C8—C7—C12	118.51 (17)	C21—C22—H22	119.2
C8—C7—C4	121.22 (18)	C17—C22—H22	119.2
C12—C7—C4	120.25 (18)	C24—C23—C28	118.57 (19)
C7—C8—C9	118.56 (18)	C24—C23—C5	121.78 (19)
C7—C8—H8	120.7	C28—C23—C5	119.65 (19)
C9—C8—H8	120.7	C25—C24—C23	120.9 (2)
N10—C9—C8	123.24 (19)	C25—C24—H24	119.6
N10—C9—N13	112.57 (16)	C23—C24—H24	119.6
C8—C9—N13	124.18 (18)	C26—C25—C24	118.4 (2)
C11—N10—C9	117.27 (16)	C26—C25—H25	120.8
N10—C11—C12	123.80 (19)	C24—C25—H25	120.8
N10—C11—H11	118.1	F2—C26—C27	119.2 (2)
C12—C11—H11	118.1	F2—C26—C25	118.0 (2)
C11—C12—C7	118.56 (19)	C27—C26—C25	122.8 (2)
C11—C12—H12	120.7	C26—C27—C28	118.4 (2)
C7—C12—H12	120.7	C26—C27—H27	120.8
C14—N13—C9	127.44 (16)	C28—C27—H27	120.8
C14—N13—H13	127.6	C27—C28—C23	121.0 (2)
C9—N13—H13	105.0	C27—C28—H28	119.5
O15—C14—N13	124.24 (19)	C23—C28—H28	119.5
			
C6A—S1—C2—C3	−27.8 (2)	C4—C7—C12—C11	−177.6 (2)
S1—C2—C3—N3A	33.4 (2)	N10—C9—N13—C14	−176.67 (19)
C2—C3—N3A—C6A	−25.1 (3)	C8—C9—N13—C14	2.2 (3)
C2—C3—N3A—C4	170.3 (2)	C9—N13—C14—O15	0.0 (4)
C6A—N3A—C4—C5	2.2 (2)	C9—N13—C14—C16	−179.99 (19)
C3—N3A—C4—C5	167.9 (3)	O15—C14—C16—C17	27.2 (3)
C6A—N3A—C4—C7	−174.4 (2)	N13—C14—C16—C17	−152.78 (19)
C3—N3A—C4—C7	−8.7 (4)	C14—C16—C17—C18	−74.3 (3)
N3A—C4—C5—N6	−1.3 (2)	C14—C16—C17—C22	100.8 (2)
C7—C4—C5—N6	174.8 (2)	C22—C17—C18—C19	0.6 (4)
N3A—C4—C5—C23	177.6 (2)	C16—C17—C18—C19	175.8 (2)
C7—C4—C5—C23	−6.3 (4)	C17—C18—C19—C20	−0.6 (4)
C4—C5—N6—C6A	−0.2 (3)	C18—C19—C20—C21	0.1 (5)
C23—C5—N6—C6A	−179.2 (2)	C18—C19—C20—F1A	−175.5 (3)
C5—N6—C6A—N3A	1.7 (3)	C18—C19—C20—F1B	172.8 (7)
C5—N6—C6A—S1	−173.1 (2)	C19—C20—C21—C22	0.3 (5)
C4—N3A—C6A—N6	−2.5 (3)	F1A—C20—C21—C22	176.3 (3)
C3—N3A—C6A—N6	−171.4 (2)	F1B—C20—C21—C22	−167.3 (12)
C4—N3A—C6A—S1	173.32 (16)	C20—C21—C22—C17	−0.2 (5)
C3—N3A—C6A—S1	4.5 (3)	C18—C17—C22—C21	−0.2 (4)
C2—S1—C6A—N6	−170.9 (3)	C16—C17—C22—C21	−175.4 (3)
C2—S1—C6A—N3A	14.27 (19)	C4—C5—C23—C24	−34.4 (3)
C5—C4—C7—C8	140.0 (2)	N6—C5—C23—C24	144.5 (2)
N3A—C4—C7—C8	−44.5 (3)	C4—C5—C23—C28	146.3 (2)
C5—C4—C7—C12	−41.6 (4)	N6—C5—C23—C28	−34.9 (3)
N3A—C4—C7—C12	133.9 (2)	C28—C23—C24—C25	−0.4 (3)
C12—C7—C8—C9	−2.5 (3)	C5—C23—C24—C25	−179.7 (2)
C4—C7—C8—C9	175.9 (2)	C23—C24—C25—C26	0.5 (3)
C7—C8—C9—N10	2.4 (3)	C24—C25—C26—F2	179.8 (2)
C7—C8—C9—N13	−176.35 (19)	C24—C25—C26—C27	0.0 (4)
C8—C9—N10—C11	−0.3 (3)	F2—C26—C27—C28	179.8 (2)
N13—C9—N10—C11	178.53 (19)	C25—C26—C27—C28	−0.4 (4)
C9—N10—C11—C12	−1.5 (3)	C26—C27—C28—C23	0.5 (3)
N10—C11—C12—C7	1.3 (3)	C24—C23—C28—C27	−0.1 (3)
C8—C7—C12—C11	0.9 (3)	C5—C23—C28—C27	179.3 (2)

### Hydrogen-bond geometry (Å, °)

**Table:**

*D*—H···*A*	*D*—H	H···*A*	*D*···*A*	*D*—H···*A*
N13—H13···N6^i^	0.97	2.02	2.980 (2)	171
C8—H8···O15	0.95	2.24	2.845 (3)	120

Symmetry codes: (i) *x*−1/2, −*y*+1/2, *z*+1/2.
